# Deep-Framework: A Distributed, Scalable, and Edge-Oriented Framework for Real-Time Analysis of Video Streams

**DOI:** 10.3390/s21124045

**Published:** 2021-06-11

**Authors:** Alessandro Sassu, Jose Francisco Saenz-Cogollo, Maurizio Agelli

**Affiliations:** Center for Advanced Studies, Research and Development in Sardinia (CRS4), Località Pixina Manna, Edificio 1, 09010 Pula, CA, Italy; jsaenz@crs4.it (J.F.S.-C.); agelli@crs4.it (M.A.)

**Keywords:** real-time video analytics, edge computing, deep learning, distributed systems, software framework

## Abstract

Edge computing is the best approach for meeting the exponential demand and the real-time requirements of many video analytics applications. Since most of the recent advances regarding the extraction of information from images and video rely on computation heavy deep learning algorithms, there is a growing need for solutions that allow the deployment and use of new models on scalable and flexible edge architectures. In this work, we present Deep-Framework, a novel open source framework for developing edge-oriented real-time video analytics applications based on deep learning. Deep-Framework has a scalable multi-stream architecture based on Docker and abstracts away from the user the complexity of cluster configuration, orchestration of services, and GPU resources allocation. It provides Python interfaces for integrating deep learning models developed with the most popular frameworks and also provides high-level APIs based on standard HTTP and WebRTC interfaces for consuming the extracted video data on clients running on browsers or any other web-based platform.

## 1. Introduction

Cities, industries, shops, and homes all around the world are experiencing a sharp increase in the number of installed video cameras for surveillance, traffic/process monitoring, and other applications that require real-time video content analysis. Traditionally, a human operator needs to manually watch camera feeds and press a button in case of an event. Since security personnel can hardly remain alert for monitoring tasks after 20 min [1], in recent years intelligent video analytics systems have emerged as a solution to analyse video streams without human intervention.

With the success of deep learning (DL), video analytics using DL is increasingly being employed to provide accurate and reliable detection, tracking, and classification of objects, people, and faces in video data. However, performing real-time and information-rich analytics using deep learning is a complex endeavor that requires not only high computational power but also a timely orchestration of several different algorithms and software modules. Most of the existing solutions for deploying DL models for video analytics rely on centralized architectures and/or cloud computing platforms hosted by large-scale IT-companies (Amazon Web Services, Google Cloud, Microsoft Azure, IBM Cloud). They provide flexible and scalable solutions with developer-friendly environments and standard interfaces but are not able to meet the Quality of Experience (QoE) demands often associated with network latency and Round Trip Time (RTT) constraints, especially for multi-camera applications.

Given the large scale of the data generated by cameras and the real-time requirements often involved, an edge solution is the most practical approach as it conducts the video analytics close to (or on) the cameras [2]. By deploying shared resources at the Internet edge and pushing computation close to the data sources, edge solutions not only benefit applications that require low latency but also those that involve privacy sensitive content. In recent years, several solutions for performing real-time video analytics at the edge have been proposed [3,4,5,6,7,8,9,10,11,12,13,14]. However, there is still a lack of contributions that can be scaled using distributed computing, that can be easily expanded with models developed with popular DL frameworks, and that can provide standard interfaces for both native and web-based client applications.

In this work, we propose a distributed and scalable software framework for implementing real-time video analytics applications named Deep-Framework (DF). Deep-Framework exploits state-of-the-art distributed technologies and allows for the implementation of DL models developed with the most popular open-source DL libraries. The framework provides a simple interface for defining and configuring the computation cluster that abstracts the complex distribution and management of resources, a high-level API for consuming the extracted video data and monitoring the system performance in an open and easy to digest format, and a web-friendly streaming interface for consuming both the resulting data and the source video on client applications running on browsers or other web-based platforms. The contribution of the proposed framework to the state of the art resides in the novel combination of the following features:

- Distributed architecture for deploying the real-time analysis of video on a cluster of small cost-effective machines as well as on a single powerful machine;
- Resource allocation mechanism that automatically allocates every algorithm according to its deep learning framework, optimizing the use of GPU and CPU resources in the cluster;
- Modular organization of components that allows efficient scaling to a large number of video cameras with multiple processing pipelines per video stream;
- Frame-skipping policy, adopted for each component of the processing pipelines, that ensures a real-time behavior of the system;
- Python interfaces that allow researchers and developers to easily include and deploy new deep learning models using the most mature deep learning frameworks;
- Standard HTTP and WebRTC APIs for providing web-based video analytics services and allowing the integration of web-based client applications.

The rest of this paper is organized as follows. Related work is discussed in Section 2, while Section 3 describes the proposed Deep-Framework in detail and explains the tools provided by the framework for integrating new models and deploying an application; Section 4 presents some experimental evaluations and describes two examples of real-world applications designed with Deep-Framework; and finally, conclusions and future work are presented in Section 6.

## 2. Related Work

Existing solutions for performing real-time video analytics at the edge can be divided between application (and model) specific systems [3,4,5,6], frame filtering strategies for reducing network usage and optimizing latency and computational resources [7,8,9,10], and general purpose architectures or software frameworks that can be used as skeletons or scaffolds for deploying models and developing applications [11,12,13,14]. Since the focus of the present paper is on the latter category, next we review with special attention the related relevant works.

Nazare and Schwartz [11] proposed Smart Surveillance Framework (SSF), one of the first general purpose real-time intelligent surveillance frameworks based on user-defined modules that communicate through a shared memory. It was built with the aim of helping researchers develop functional surveillance systems, as well as integrate new algorithms to solve problems related to video surveillance. SSF provided a set of mechanisms for data representation, storage, and communication and also provided tools for performing scene understanding. Although it allowed for the integration of modules written in C++ in a flexible way, it was a single camera/stream solution designed to be deployed on a standalone machine and was not optimized for executing DL models. In [12], Ali et al., proposed a system for analyzing multiple video streams with DL models distributed between the edge and the cloud. Its aim was to bring the deep learning based analytics towards the source of the video streams by parallelizing and filtering streams on the network nodes. It implements a background filtering approach for filtering background frames in in-transit nodes, which help to reduce the video data, bandwidth, and the storage needed at the destination cloud. It demonstrated the throughput gain of not executing models entirely on the cloud but it did not provide any tool for configuring the system, integrating new models, or for easily consuming the resulting data. Rouhani et al. [15] presented a framework for real-time background subtraction on FPGA called RISE. The goal of this framework is to provide a user-friendly interface that can be used for rapid prototyping and deployment of different video surveillance applications using SoC platforms. RISE takes the stream of a surveillance video as its input and learns/updates a fixed-size dictionary matrix as a subsample of the input pixels. The dictionary samples are selected to capture the varying background pixels in the input video. Though RISE is presented as a multipurpose framework, it is limited to embedded systems and its API only allows for the customization of the parameters of a single background subtraction algorithm. Liu et al. [13] introduced EdgeEye, a service-oriented framework based on GStreamer that provides a high-level, task-specific API for developing and deploying real-time video analytics applications. The aim of EdgeEye is to bring computation entirely at the Internet edge, focusing on applications that require low latency and high privacy. The API allows to load and manage predefined DL models and video and data can be consumed via WebRTC-based peer-to-peer connections. Though this approach represents a flexible and ‘user-friendly’ solution it does not provide an interface for integrating user-defined models and relies on a single machine for performing all the processing work, thus limiting the application to lightweight models, few camera streams, or powerful machines with multiple GPUs. In [14], Uddin et al., presented a distributed video analytics framework that allows one to process both real-time streams and batch video using Apache Kafka for capturing video frames from multiple sources and Apache Spark for the distributed computing of video streams. The aim of this framework was to provide a complete service-oriented ecosystem focusing on scalability, effectiveness, and fault-tolerance. It consists of five main layers, Big Data Curation Layer, Distributed Video Data Processing Layer, Distributed Video Data Mining Layer, Knowledge Curation Layer and Service Curation Layer. Moreover, it has three basic users with roles: Administrator, who provides and manages the platform, Third Party Developers, who can use the existing video processing APIs provided by the framework or can develop new APIs to produce new services, and End-Users, who can use the existing services. Though it provides a Java-based service-oriented ecosystem with specific interfaces for end-users and developers, it does not provide an interface for integrating user-defined models. None of the mentioned works enforce a frame-skipping policy for ensuring the real-time processing of video streams or simple interfaces for both developing a complete video analytics application and integrating new DL models built with popular Python DL frameworks.

## 3. Materials and Methods

Deep-Framework is designed as a software framework to enable DL researchers and developers rapid prototyping of distributed, scalable, multi-algorithm, and latency-focused solutions in edge computing contexts. The methods and strategies adopted for developing the framework are chosen with two types of users in mind: application developers and DL DevOps engineers. The idea is that application developers can implement, configure, and use the framework codebase and integrated models to quickly deploy a complete real-time video analytics backend focusing on the end-user application and data consumption without requiring to handle the orchestration of services and model integration. On the other hand, the framework allows DL DevOps engineers to focus on the integration of new DL models simplifying the creation of processing pipelines and the deployment of a test application.

Being a distributed edge framework, Deep-Framework is designed to be executed in a cluster of computational nodes. Each component of the framework works as an independent service that is deployed in one of the nodes as a Docker service in swarm mode [16]. Each service is scheduled on a node based on specific rules that depend on the configuration desired by the user. They communicate with each other through TCP sockets using ZeroMQ [17] taking advantage of its predefined messaging-patterns that allow scalability in distributed systems with almost zero overhead [17,18]. The docker images are created in a single main node and are then distributed, via a docker registry, to the remaining nodes in a transparent manner to the user, who only needs to provide the IP address and the credentials of the machines.

**Figure 1:**
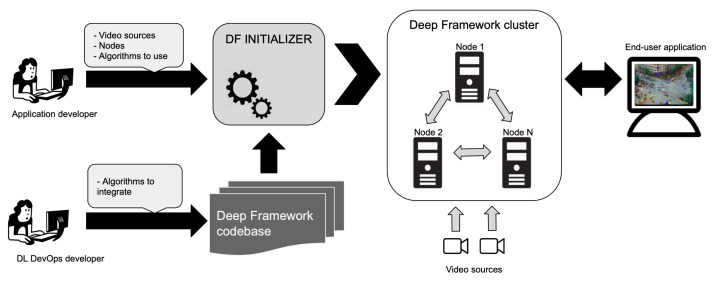
Overview of Deep-Framework workflow. The framework provides tools for the integration of new algorithms and for defining and launching a complete video analytics application on a computational cluster based on available models.

The creation and deployment of processing pipelines and the orchestration of all services is managed by a component called DF Initializer (as depicted in Figure 1), which provides a command-line interface that allows users to define the video sources, to specify the available computation nodes, and to choose the algorithms (models) that intend to execute. With this information the DF Initializer performs the following actions:

- Initializes the components, builds the pipelines, and creates the related services;
- Creates the cluster of nodes in which the services will be executed;
- Allocates the services with DL models that require GPU to nodes with GPU capability, taking into account the number of nodes, the available memory, and the installed DL frameworks; the other services are scheduled on a different node whenever possible. In Algorithm 1, this procedure is explained in detail;
- Defines the connection with video sources.

**Algorithm 1:** Allocation of algorithm services to nodes.

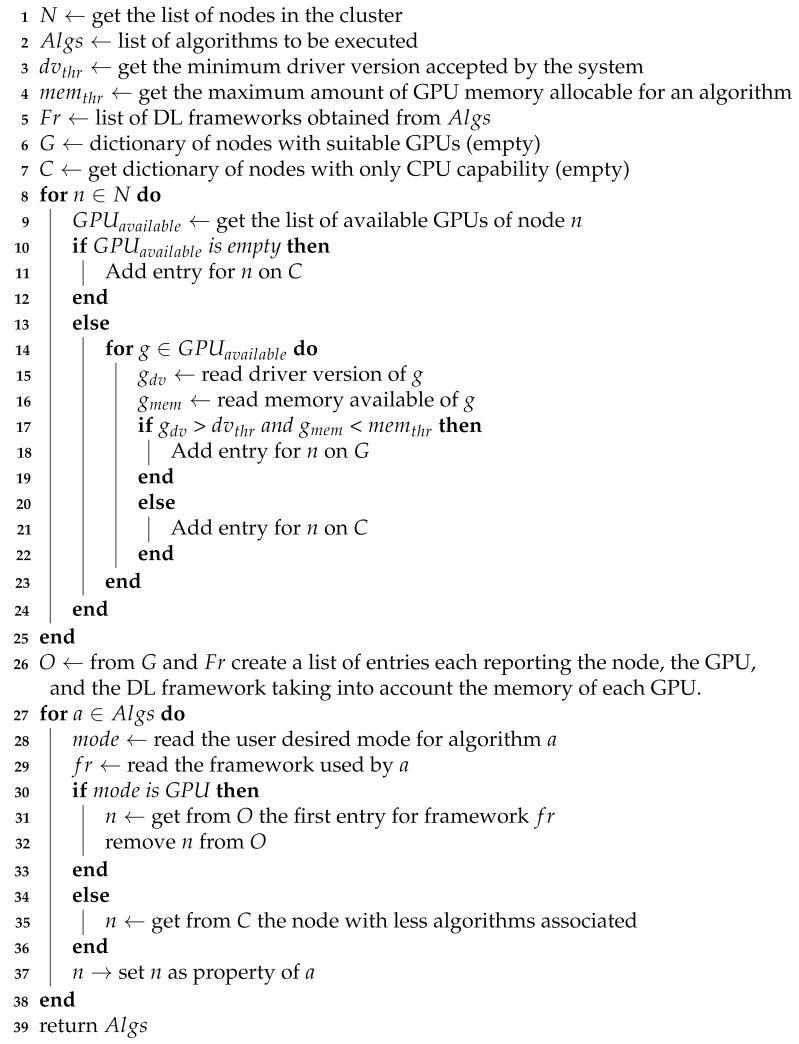



### 3.1. Architecture

**Figure 2:**
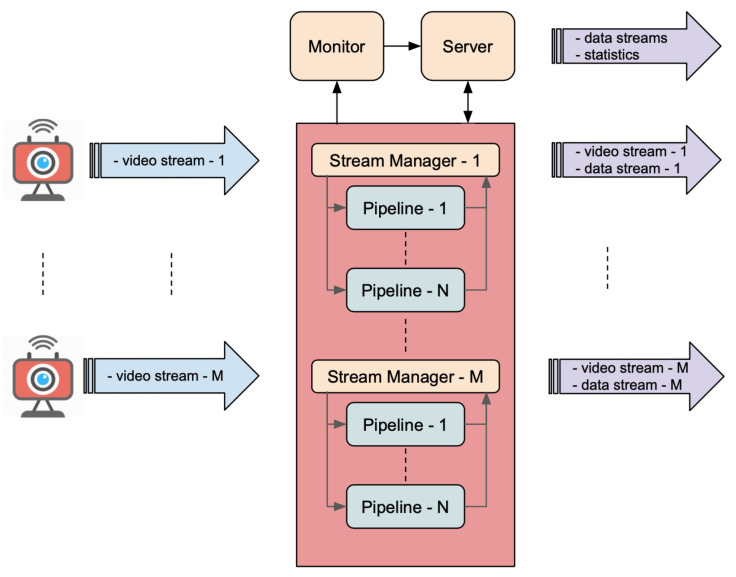
Functional architecture of Deep-Framework. The system is able to process multiple video streams at once through one or more pipelines that are responsible for information extraction.

As shown in Figure 2, the framework manages each video stream independently through a Stream Manager module which distributes individual frames to one or more processing pipelines, that parallelize all video analysis tasks, and is able to stream out both the resulting data stream and the input video stream via peer-to-peer connections. A Server module provides the API for consuming the resulting data, managing the stream processing, and monitoring the performance of the individual components thanks to a dedicated Monitor component.

Each processing pipeline detects and analyses a different type of object or Region of Interest. A pipeline performs the following tasks:

- *Object detection*: objects or areas of interest within an image are identified and tracked along subsequent images using detection and tracking methods. The component that performs this task is called Detector.
- *Object description*: objects are classified, characterized, or described using classification models, feature extractors, or generic analytical models. These tasks are performed by three components: Broker, Descriptor, Sub Collector. These components form a Descriptor Chain. Several Descriptor Chains may operate in parallel, each one dedicated to the extraction of a specific feature.
- *Results collection*: results are collected and aggregated to produce the output data stream. The component that performs this action is called Collector.

#### 3.1.1. Processing Pipeline

**Figure 3:**
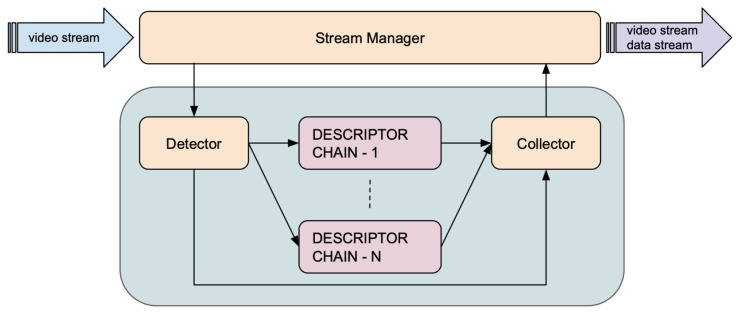
Processing pipeline. A processing pipeline is composed essentially of: at least one Detector, one or more Descriptor Chain (not mandatory), one for each feature to be extracted, and a Collector.

**Figure 4:**
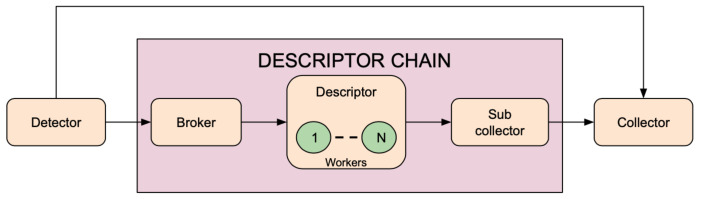
Components of a pipeline Descriptor Chain. A Descriptor Chain allows a scalable method of feature extraction distributing the messages coming from Detector across the N Descriptor workers. The Broker and the Sub Collector act, respectively, as a producer and a result collector in a push/pull messaging pattern.

As shown in Figure 3, a processing pipeline is composed essentially of: at least one Detector, one or more Descriptor Chains (not mandatory), one for each feature to be extracted and a Collector. A Descriptor Chain is composed of Broker, Descriptor, and Sub Collector (as illustrated in Figure 4). Next, every component is described in detail.

#### Detector

This component is responsible for extracting the coordinates of an object in an image and for tracking it along all received images. The object could be an entity that is depicted in a particular region of the image or in the entire image. Once the coordinates of the object have been estimated, they are sent, together with the corresponding Region of Interest (ROI), to the components dedicated to the information extraction. The framework provides a Python abstract class that defines a common interface for allowing the integration of custom detection and tracking models. In this way, developers only have to deal with the analysis of images and inference-related operations. Each Detector defines a different pipeline. The framework, therefore, wraps the custom code and performs functions as follows:

- It instantiates the desired Detector;
- It receives images and enforces the frame skipping mechanism (as described below in this section);
- It creates a list of objects, each composed of an identifier, the coordinates of the bounding box, and and/or a list of keypoints, if available, and sends them to the Collector component;
- It sends the list of objects and their ROIs to the connected descriptors, using a publish–subscribe messaging pattern.

Due to its fundamental function, the Detector gets priority in memory allocation procedure performed by the DF Initializer and described in Algorithm 1.

#### Descriptor

This is the component that carries out the analysis of the ROIs and/or coordinates, retrieved by the Detector, with the aim of extracting information about objects and/or their trajectories. In order to scale up this process, it is possible to create several instances (workers) for each Descriptor. Similar to the Detector, the DF provides a Python abstract class that defines a common interface for the integration of custom models. The framework, therefore, wraps the custom code and performs functions as follows:

- It instantiates the desired Descriptor;
- It receives images and enforces the frame skipping mechanism;
- It extracts feature from ROIs and performs an average of the results obtained on N successive images in which the object is tracked.

#### Broker

This component receives data from the detector and distributes them across all the instances of the Descriptor.

#### Sub Collector

It aggregates the results obtained by the workers instantiated for each Descriptor; only the most recent results are sent to the main Collector.

#### Collector

For every result coming from Detector (objects coordinates, identifiers), it produces an output message aggregating the latest available results obtained from Sub Collectors (features) and the timestamps of the corresponding frames. The algorithm used by Collector to aggregate data and construct the output message is shown in Algorithm 2. Note that retrieving results from Descriptor Chains is a not blocking operation and that, if no new results are available from a specific Descriptor, the latest ones are aggregated. Therefore, though each output message contains results from all running algorithms, each result may be related to a different frame and thus be associated to a different timestamp. The resulting data stream is made available to the components that implement the streaming APIs.
**Algorithm 2:** Collector data aggregation and output message creation algorithm.
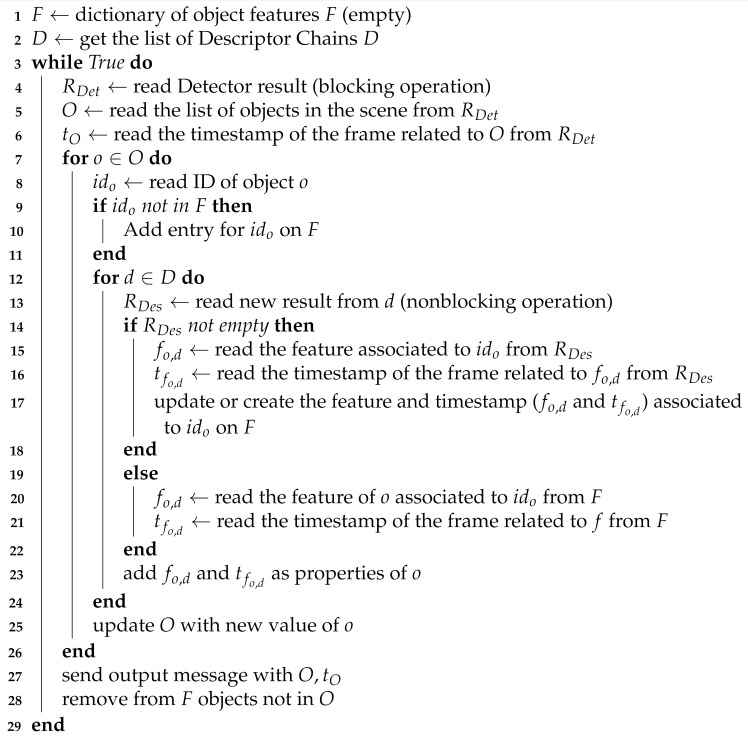


Parallelization of video analysis tasks is therefore achieved at 3 different levels: (1) routing the video stream to different pipelines, each one dedicated to the analysis of a specific type of objects or ROI, (2) extracting in parallel different features through dedicated Descriptor Chains, and (3) distributing across several workers the feature extracting tasks of each Descriptor.

Regardless of the amount of computational resources allocated, real-time behavior is ensured by a frame-skipping algorithm, implemented in the Detector and the Descriptor, which basically consists in skipping the frames received during the previous computation and analysing only the most recent one.

The DF is intended as a feature extractor engine, so none of the above components store anything permanently. In fact, in DF, the images received by the components remain in memory for the time needed by the computation operations. The video and the data resulting from the analysis can be possibly stored by an external application.

#### 3.1.2. Stream Manager

The Stream Manager establishes the connection with the video source in order to grab the individual frames and send them, together with a timestamp, to the processing components. Sources can be RTSP/HTTP streams (IP cameras), WebRTC streams (web-based video providers), or video files (for non-real-time applications or testing purposes). The DF can process multiple sources at once. For every source a Stream Manager instance is created. It also receives the resulting data from the Collector component and is able to stream it together with the input video stream through a WebRTC connection mediated by the Server. Stream Manager and all pipeline components are implemented in Python.

#### 3.1.3. Server

The Server provides an HTTP API for distributing resulting data streams, sending statistics about the performance of individual components, controlling the execution of pipelines, and listing the available resources. Data streams, received from Collectors, are distributed through endpoints based on Server-Sent Events (SSE). Statistics are also distributed through SSE but are collected from a dedicated Monitor component that will be described in the following section. The control of pipeline execution is exerted through connections with Stream Managers and allows one to start or stop the grabbing of frames from a video source. The Server also acts as a WebRTC signaling server based on Websockets and allows client applications to establish peer-to-peer connections with single Stream Managers for receiving both video and data streams. Since WebRTC connections are bidirectional, this kind of communication allows external applications to also act as a video source, thus opening the possibility for clients or remote agents to use a single connection for both sending the video stream and receiving the resulting data. The Server is implemented in Node.js.

#### 3.1.4. Monitor

The Monitor is connected with all pipeline components and receives and aggregates from them their operating metrics, which include the throughput (results per second) and the number of frames received, processed, and skipped. These metrics, once collected and aggregated, are made available through the Server and can be used to evaluate the performance of a specific component and of the whole system.

### 3.2. Using Deep-Framework

Deep-Framework, as described above, can be used from application developers and DL DevOps engineers. Application developers can use the framework by interacting with DF Initializer in order to configure and deploy the application services using already defined Detectors and Descriptors (for instance those used in Section 4.2, which are included in the framework codebase repository). DF Initializer allows users to specify via the command line interface the following information:

- Cluster data (number of nodes and their type, IP addresses, user credentials);
- Frequency for generating statistics;
- Video sources and their types (video file, IP stream, WebRTC stream);
- Detectors to use (among those available) for each video source along with their computation mode (CPU/GPU).
- Descriptors to use (among those available) for each detector (pipeline) along with their computation mode (CPU/GPU) and number of instances (workers).

DL DevOps engineers can also develop and include their own algorithms among Detectors or Descriptors. The procedure to integrate a custom algorithm is quite similar in both cases and will be described in the following sections and, more in detail, in Appendix A.

#### 3.2.1. Integrating a Custom Detector

**Figure 5:**
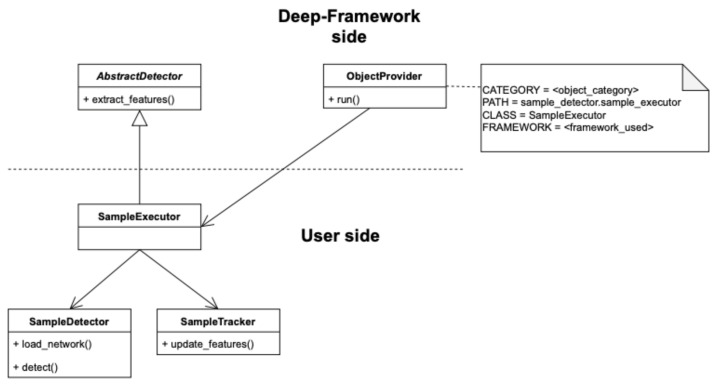
Detector class diagram showing the relation between the Deep-Framework classes and the user-defined classes related to the Detector component. More details are provided in Appendix A.1.

The interface for integrating custom detection and tracking algorithms is provided through a Python abstract class called AbstractDetector as illustrated in Figure 5. For example, the custom detector and tracker classes, named in the Figure 5 SampleDetector and SampleTracker, implement the detection and tracking techniques chosen by the user. The SampleExecutor class extends the AbstractDetector class and implements the extract_features method wrapping these classes and using their methods in order to provide the features of interest along subsequent frames. The ObjectProvider class, on the platform side, dynamically creates an instance of the executor chosen by the user taking as input the corresponding configuration file. More details are provided in Appendix A.1.

#### 3.2.2. Integrating a Custom Descriptor

**Figure 6:**
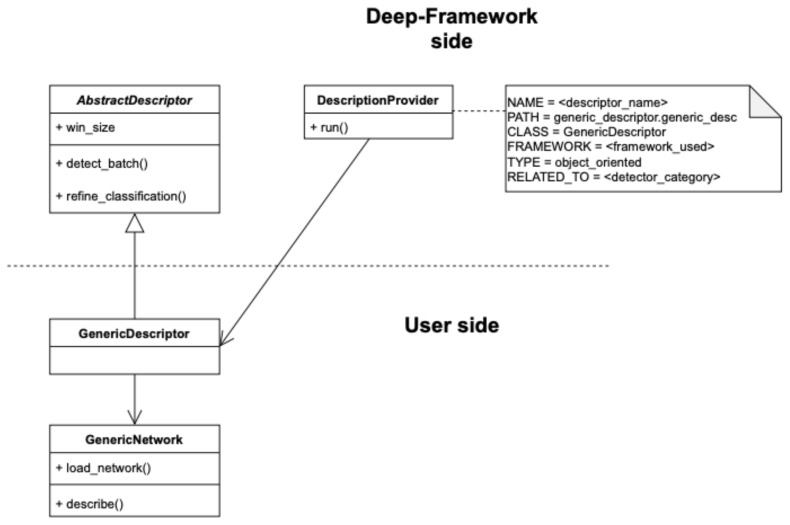
Descriptor class diagram, showing the relation between the Deep-Framework classes and the user-defined classes related to the Descriptor component.

Similarly to the integration of a custom Detector, the interface for integrating custom object description algorithms is provided through a Python abstract class called AbstractDescriptor. As shown in Figure 6, GenericNetwork may be the class that implements the algorithm of feature extraction. This class can be wrapped by a custom Descriptor GenericDescriptor class that extends AbstractDescriptor and implements the detect_batch method, which provides the classification result of the objects list received by the Detector, obtained on a single frame, and the refine_classification method, which should return as output the results averaged over a number of frames specified by the win_size variable. The DescriptionProvider class, on the Deep-Framework side, dynamically creates an instance of the Descriptor chosen by the user taking as input the corresponding configuration file. More details are provided in Appendix A.2.

## 4. Results

### 4.1. Experimental Results

In order to evaluate the performance and scalability of the framework, we conducted two experiments aimed at evaluating the behavior of the system when scaling up a single Descriptor chain (increasing the number of Descriptor workers) and the computational cluster (increasing the number of nodes) for a constant load task. Two computers with the following characteristics are used:

- PC1: OS Ubuntu 16.04 LTS, processor Intel(R) Xeon(R) CPU E5-2630 v4 @ 2.20 GHz with 20 cores, 64 GB RAM, NVIDIA GeForce GTX 1080 Ti;
- PC2: OS Ubuntu 20.04 LTS, processor Intel(R) Core(TM) i9-9900K CPU @ 3.60 GHz with 16 cores, 32 GB RAM, NVIDIA GeForce RTX 2080 Ti.

In the first experiment, a video with 24 fps, generated from a static image of size 1280 × 720 and containing four equal crops 151 × 176 of a face, was analyzed. The framework was deployed on a single node (PC1) with a single pipeline composed of a Detector based on MTCNN [19] and optical flow and a single Descriptor based on a popular gender estimation model [20]. We performed this test both in CPU and GPU mode (only for Descriptor) with an increasing number of Descriptor workers while monitoring the resulting performance in terms of Descriptor throughput and system throughput, averaged over 100 frames. The Descriptor throughput represents the frequency to which the features of each detected object are updated, whereas the system throughput is the frequency of the output messages produced by the Collector which produces one message for every result coming from Detector.

**Figure 7:**
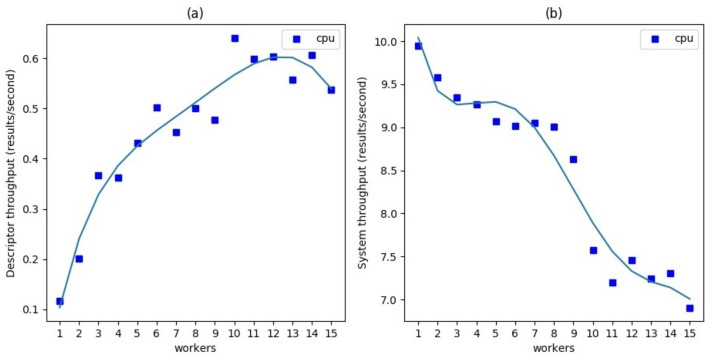
Scalability of a single Descriptor chain increasing the number of workers in CPU mode. (**a**) Descriptor throughput. It indicates the number of feature updates per second. (**b**) System throughput. It indicates the number of output messages per second.

Figure 7a shows that, when using CPU mode, Descriptor throughput increases significantly in an almost linear way with the number of workers. Since in this case all services are in a single node, increasing the number of Descriptor workers also decreases the performance of the Detector, which results in a reduced system throughput as is shown in Figure 7b. However, this reduction is quite limited in comparison with the gain observed in the Descriptor throughput: when six workers are used, there were nearly 5× times faster Descriptor updates with only a 10% reduction in system throughput. After a certain limit (eight workers in this case), increases in the number of workers produce a sharp decline in system performance, probably due the exhaustion of the machine computational resources. In this experiment the Detector does not represent a bottleneck, its response time being negligible (~100 ms) compared to the response time of a single Descriptor (~10 s). In this case, distributing the Descriptors across several workers can sensibly increment the Descriptor throughput.

**Figure 8:**
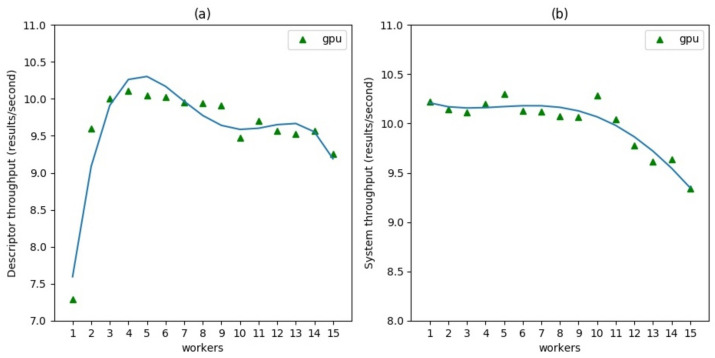
Scalability of a single Descriptor chain increasing the number of workers in GPU mode. (**a**) Descriptor throughput. It indicates the number of feature updates per second. (**b**) System throughput. It indicates the number of output messages per second.

Figure 8 shows the results of the same experiment in GPU mode. This time the gain scaling on several workers slightly improves the Descriptor throughput up to about 30% with three workers, above which the throughput reaches a plateau very close to the value of system throughput which indicates that Descriptors manage to process all Detector results. The overall system throughput shows an essentially regular pattern before decreasing from worker number 12 and beyond, due to the machine reaching its operational capability limits.

**Figure 9:**
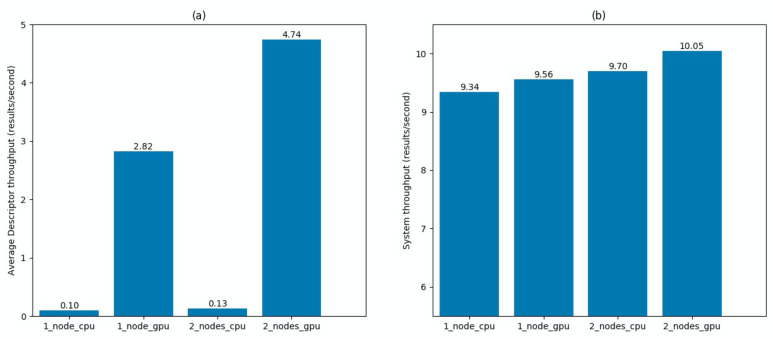
Scalability of six identical Descriptor chains using one or two nodes in both CPU and GPU modes. (**a**) Average Descriptor throughput. It indicates the number of feature updates per second averaged among the six Descriptor chains. (**b**) System throughput. It indicates the number of output messages per second.

For the second experiment, the same video stream was analyzed with the same Detector, but this time using six different Descriptors with equal computational complexity (i.e., running on each the same gender estimation algorithm). We measured the average Descriptor throughput and the system throughput in four system configurations: one node in CPU mode, one node in GPU mode, two nodes in CPU mode, and two nodes in GPU mode. PC1 was used for one node measurements and both PC1 and PC2 for two node measurements. All Descriptors were deployed with one worker. The results are shown in Figure 9. Distributing the application on two computational nodes produced an improvement of Descriptor throughput of 30% in CPU mode and of 68% in GPU mode. The improvement of the system throughput, being related to the Detector performance which runs in all cases in CPU mode, shows only a small increase due to the indirect effect of a better distribution of resources.

### 4.2. Application Examples

In this section, the design and implementation of two real-world applications using Deep-Framework is described. The development of these applications intends to demonstrate the usefulness and flexibility of Deep-Framework as a tool for designing real-time video analytics solutions using state-of-the-art DL models. Since the goal is to show how these applications can be developed with Deep-Framework, details of the adopted DL models are not presented. In order to monitor the performance and visualize the results of both applications, a demo web application, based on AngularJS, was developed to show the extracted data overlaid on video in real-time. The performance results are summarized for the two applications and evaluated in terms of the respective real-time requirements.

#### 4.2.1. Customer Intelligence Application

**Figure 10:**
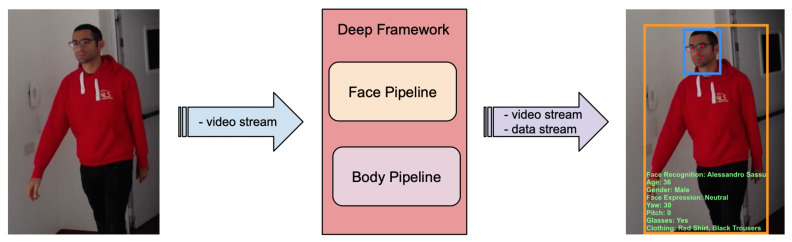
Example of a customer intelligence application designed with Deep-Framework.

The goal of this application is to use a camera feed to characterize the customers of a clothing shop. The idea is to extract customer information at both face and body levels. From face analysis the application recognizes registered customers, estimates age and gender, estimates gaze direction and face emotive expression, and detects whether customers wear glasses or not. From body analysis the application recognizes the clothing type. The resulting data can be used for statistical analysis and triggering messages to shop assistants whenever some specific conditions are met. As shown in Figure 10, this type of application naturally defines a two-pipeline solution using Deep-Framework.

**Figure 11:**
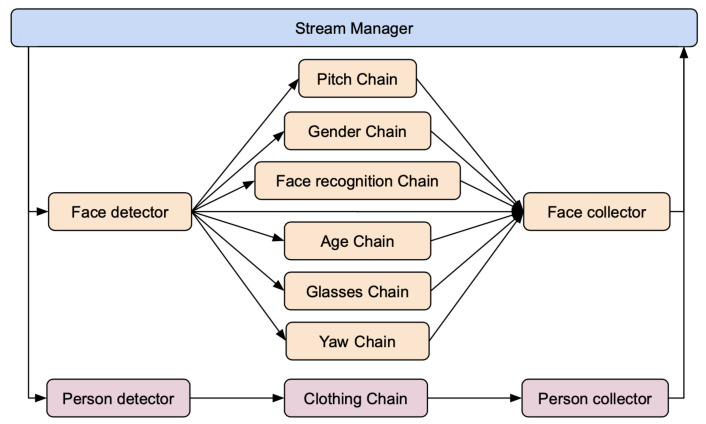
Composition of the processing pipelines for the customer intelligence application.

In the Detector of the face pipeline, a face detection and tracking approach is implemented based on the combination of MTCNN [19] and optical flow. Briefly, faces are first detected in images using MTCNN which output their bounding boxes and a set of facial landmarks that are then tracked through successive images with the OpenCV implementation of the optical flow-based Lukas–Kanade method. This approach allows for the detection of faces faster than using the MTCNN model on each image. The next section of the face pipeline is composed of seven Descriptor Chains; in each of them a specific DL model is implemented for the extraction of the required information using the following algorithms: age estimation [20], gender estimation [20], face recognition based on [21], glasses detection based on [22], face expression recognition based on [23], yaw and pitch estimation [24]. In the Detector of the body pipeline, an object detector based on MobileNet-SSD [25] and a tracker based on [26] were used. A model based on [22] was used for the classification of the clothing type. Figure 11 illustrates the composition of the processing pipelines.

In order to demonstrate the performance of the DF in this example application, the proposed solution was deployed on a cluster of two nodes (PC1 and PC2 as described in Section 4.1) and the Descriptor and the system throughput were measured while analyzing a video that captured one person as in Figure 10. Since in this case results are produced by two independent pipelines, the system throughput is actually described by the individual pipeline throughputs which, as already explained, depend on the frequency of Detectors. Both Detectors (face and body) were deployed in CPU mode, while the eight Descriptors (yaw, pitch, glasses, face recognition, clothing, age, gender, emotion), each of them with one worker, were deployed in GPU mode. Table 1 summarizes the results. Note that most Descriptors have a throughput close to that of their respective pipeline which means that they manage to process most of the frames processed by the Detector.

Considering that the aim of this application is to collect customer information which typically does not change considerably during an observation period of a few seconds, the measured throughput is appreciably above the expected system capacity. Moreover, assuming a response time of 1 s is sufficient for alerting a shop assistant when a specific event occurs, real-time requirements are largely met. With many people/faces detected at the same time or with more computation demanding descriptors, it may be necessary to allocate more workers for extracting those features which are expected to change more rapidly.

#### 4.2.2. Crowd/Traffic Monitoring Application

**Figure 12:**
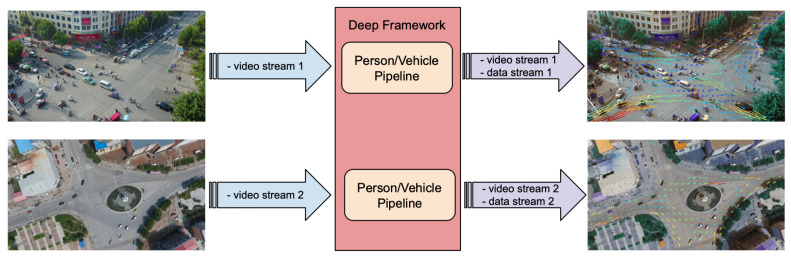
Example of a crowd/traffic monitoring application designed with Deep-Framework.

**Figure 13:**
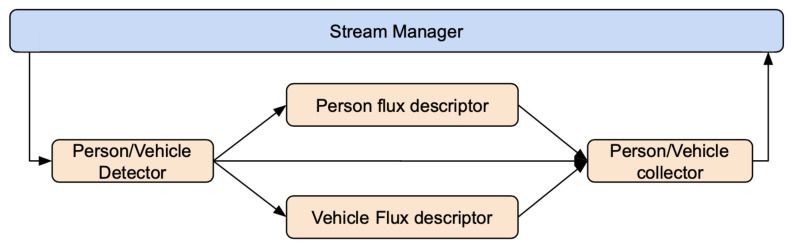
Composition of the processing pipeline for the crowd/traffic monitoring application.

In this application the goal is to monitor the movements of pedestrians and vehicles at critical and/or strategic locations of an urban scenario. The idea is to extract the trajectories of pedestrians and vehicles and also to characterize the scene captured by each camera in terms of flux, velocity/speed, and occupation of each type of object (as depicted in Figure 12). In order to implement this application with Deep-Framework, a single pipeline per video stream was defined using a Detector for identifying, tracking, and labeling objects and two Descriptors for analyzing the distribution of persons and vehicles over the captured area (Figure 13). In the Detector component, YOLOv4 [27] was employed in order to detect pedestrians, two wheeled vehicles, small four wheeled vehicles and large four wheeled vehicles, and a MOT approach based on Deep SORT [28] in order to track them. For the Descriptors, a simple algorithm is implemented that divides the image into several small square segments of equal size and calculates the average flux, velocity/speed, and occupation of all the objects that cross each segment over a fixed period of time.

As done in the Section 4.2.1, some measurements were conducted in order to evaluate the performance of the system. For this application, only PC2, whose characteristics are described in the Section 4.1, was used. The measurements, performed with one worker for each Descriptor (vehicle flux analysis and person flux analysis), revealed a system throughput of 3.07. As depicted in the Table 2, all Descriptors obtained the same throughput because they could process at most the same number of frames as the Detector, whose throughput represents the maximum value obtainable from any component.

In this application scenario the system throughput is largely limited by the detection of the many vehicles and pedestrians. However, considering that results can be collected approximately every 300 ms and that a vehicle running at 100 km/h covers a distance of about 9 m in this interval, real-time requirements should be met for a camera coverage similar to the one depicted in Figure 12.

## 5. Discussion

We have presented Deep-Framework, a software framework for developing real-time video analytics applications in edge computing contexts. DF represents a new open source tool for allowing DL researchers and developers rapid prototyping of distributed, scalable, multi-algorithm, multi-stream, web-ready, and latency-focused solutions.

DF exploits the lightweight virtualization and service orchestration of Docker containers and Docker swarm which have demonstrated to be a powerful solution for modularizing, parallelizing, and distributing analytics operations at the edge with minimal overhead [29,30]. Going beyond simply using containers and services, here we propose a tool for abstracting away from researchers and developers the complexities of resource allocation and cluster management. Moreover, the only platform requirement for building the cluster is to have machines that are visible on the network. A local docker registry is used in the main node to distribute docker images to the remaining nodes in the cluster.

At its core, DF handles the analysis of a video stream into independent processing pipelines where DL-based information extraction algorithms can be shaped using two types of components (Detector and Descriptor), which characterize the two stages of the processing pipeline. The Detector identifies (and may also classify) objects and regions of interests that are subsequently passed to Descriptors to extract additional specific features. As shown in Section 4.2 (example applications), multiple Detectors allow for the scaling up of the processing of multiple types of objects with multiple pipelines, while multiple Descriptors allow a pipeline to scale up the extraction of multiple features. Along with the possibility of executing different algorithms in parallel, the framework can run multiple instances or workers of the same Descriptor for increasing its throughput. This was explored in Section 4.1 where experiments show that for a given task in CPU mode this feature allows for the achievement of up to 5× times faster Descriptor updates before additional workers start to sensibly reduce the overall system performance. Selectively increasing the number of Descriptor workers can be a throttle that can be used for increasing the performance of an application by tuning the latency of most demanding feature extraction tasks on the basis of available resources.

Different to other works that report the development of model-specific solutions [3,4,5,6], DF not only proposes a general purpose architecture but also provides a model and application agnostic Python-based scaffold that is able to wrap deep learning models developed with the most popular and mature Python libraries leveraging on their power to utilize multi-CPU or multi-GPU hardware configurations. DF is better seen as a software framework for rapid-prototyping of applications that require real-time analysis of video streams. To the best of our knowledge there are very few works that report this kind of solution. The Smart Surveillance Framework (SSF) proposed by Nazare and Shwart [11] is similar in spirit to DF, and though it allows the creation of complex pipelines with user-defined modules thanks to a memory sharing mechanism, it does not offer the possibility to distribute the computation over a cluster or manage the allocation of computational resources taking into account the availability of CPUs and GPUs. Moreover, it does not offer the possibility to exploit the use of DL tools or libraries, which can be important for researchers and developers interested in rapidly deploying modern DL models and testing applications. Another framework that is specifically proposed for using DL models at the edge is EdgeEye [13], which, similarly to DF, provides high-level HTTP and WebRTC APIs for using integrated models and consuming the output data stream. EdgeEye inherits from GStreamer its capability to reuse many of its open-source plugins and, though it allows the integration of DL models by means of specific inference engines like TensorRT, it does not provide an interface for directly integrating models using the popular Python-based DL libraries like Tensorflow or PyTorch. More importantly, EdgeEye does not exploit distributed computing and relies on having a single powerful machine, which is not always feasible or practical in edge scenarios. In contrast, DF proposes a solution for deploying applications over a cluster of cost-effective machines as well as on a single powerful machine with multiple GPUs. In this regard, DF can be compared with the distributed video analytics framework SIAT [14], but being based on a Java enterprise-oriented ecosystem and focusing on data persistence and mining, SIAT represents more a cloud centralized solution than an edge alternative.

DF is designed for processing live video streams by implementing a frame-skipping policy which ensures a real-time behavior by always processing the latest available frame. The data stream resulting from a processing pipeline provides an output message for each frame analyzed by the Detector which determines the latency of the individuated objects or ROIs. On the other hand, the latency of each extracted feature strictly depends on the specificity of each Descriptor, which is in turn determined by the complexity of the inference model and the number of workers involved. For those reasons, not only the output stream will not contain an output message for each frame of the input stream, but not all features in an output message will be updated. This implies that a given output message may contain information about different frames. However, the associated timestamps, which are assigned by the Stream Manager at the moment of grabbing (see Section 3.1.2), always allow linking the output results to a specific input frame. Moreover, considering that the delay of the video communication can be easily measured experimentally, a client application can link the output messages to external events detected by the camera and synchronize the results from different cameras.

## 6. Conclusions

In this paper, we present a novel edge-oriented framework to develop real-time multi-stream video analytics applications based on deep learning models. Deep-Framework allows the distribution of the computing effort among one or several machines with almost zero user configuration effort, automatizing the creation of the computation cluster and allocation of GPU resources. The framework has a scalable multi-stream architecture based on Docker services where each video feed can be independently processed by multiple processing pipelines, each of them able to execute several algorithms with multiple instances each. For deep learning DevOps engineers the framework provides a Python interface that allows the integration and deployment of models using the most deep learning popular frameworks. By providing standard HTTP and WebRTC APIs, Deep-Framework allows application developers to easily consume the resulting data and video streams with desktop, mobile, or browser clients.

The experiments show how the scalability of the framework allows applications to fully exploit the computational resources available both at single-machine level and at cluster level in order to deliver timely results. Two different example applications that demonstrate the capability, scalability, and flexibility of the framework on real-world scenarios are also presented.

Deep-Framework is an ongoing open-source project; those interested in learning more or using it in their own work can find more information and the source code at https://github.com/crs4/deep_framework (accessed on 10 June 2021). Finally, Deep-Framework is under active development; feel free to suggest features at https://github.com/crs4/deep_framework/issues (accessed on 10 June 2021) so that we can continue to improve this tool for the deep learning video analytics community.

## Figures and Tables

**Table 1:** Descriptor throughput and pipeline throughput resulting for the customer intelligence application measurements obtained deploying the system in a cluster of two nodes and one worker for each Descriptor.

Descriptor Name	Throughput (Results/second)
yaw	13.18
face recognition	13.22
glasses	13.48
age	12.5
gender	12.3
emotion	12.95
pitch	13.47
clothing	22.57
**Pipeline Name**	**Throughput (Results/second)**
Person	22.63
Face	13.55

**Table 2:** Descriptor throughput resulting for the crowd/traffic monitoring application experiment conducted on a single node, with one worker for vehicle flux analysis and one for person flux analysis Descriptors.

Descriptor Name	Throughput (Results/second)
vehicle flux analysis	3.05
person flux analysis	3.05
**System throughput**	3.07

## Data Availability

All models and computer code used during this study for obtaining the presented results are available in the following public repository: https://github.com/crs4/deep_framework (accessed on 10 June 2021).

## Appendix A

### Appendix A.1. Integrating a Custom Detector

The interface for integrating custom detection and tracking algorithms is provided through a Python abstract class called AbstractDetector. The easiest way to start is by cloning the sample_detector folder in the /detector/object_extractors path inside the framework repo and renaming it with the name of the new custom detector. The code inside a very simple detector executor would look something like:

  fromutils.featuresimport Object, Rect, Point

  fromutils.abstract.detectorimport AbstractDetector

  from.detectorimport SampleDetector

  from.trackerimport SampleTracker

 classSampleExecutor(AbstractDetector):

   def__ init__(self):

     self.detector = SampleDetector()

     self.tracker = SampleTracker()

   defextract_features(self,current_frame,executor_dict):

     detected_objects =self.detector.detect(current_frame)

     tracked_objects =self.tracker.update(detected_objects)

     return tracked_objects

Note that the actual implementation of the custom detection and tracking algorithms should be done inside the SampleDetector and SampleTracker modules. The framework knows where it can find the custom code by looking at the configuration file inside the folder which looks like this:

 CATEGORY= sample_category

 PATH=sample_detector.sample_executor

 CLASS=SampleExecutor

 FRAMEWORK=tensorflow

Note that it is necessary to specify the type of DL framework used by the custom algorithms (Tensorflow in the example); this allows the framework to better allocate the GPU resources. All the dependencies and environment requirements of the custom code should be included in Dockerfiles, one per processing mode (CPU/GPU). The custom Detector will be available to be included in the video processing application at the next execution of the DF Initializer.

### Appendix A.2. Integrating a Custom Descriptor

Similarly to the integration of a custom Detector, the interface for integrating custom object description algorithms is provided through a Python abstract class called AbstractDescriptor. As before, the easiest way to start is by cloning a sample folder, this time the generic_descriptor folder in the /descriptor/feature_extractors path inside the framework repo and renaming it with the name of the new custom Descriptor. The code inside a very simple descriptor would look something like:

 from.generic_networkimport GenericNet

 fromutils.abstract_descriptorimport AbstractDescriptor

 classGenericDescriptor(AbstractDescriptor):

   def__init__(self):

     self.net = GenericNet()

   win_size  = 10

   defdetect_batch(selfdetector_results,images):

     inference_results =self.net.classify(images)

     return inference_results

   defrefine_classification(self,class_results):

     return class_results.mean()

Note that the abstract class allows for the implementation of two methods (detect_batch and refine_classification) and one class variable (win_size). On one hand, the detect_batch method receives the objects individuated by the Detector and their ROIs and should return the classification result. The refine_classification method, on the other hand, should return as output the results averaged over a number of frames specified by the win_size variable. The configuration file for defining the Descriptor and telling the framework the location of the custom code may look like this:

 NAME=generic

 PATH=genericdescriptor.generic_de

 CLASS=GenericDescriptor

 FRAMEWORK=tensorflow

 TYPE= objectoriented

 RELATED_TO= samplecategory

Note that compared with Detector settings, this time two additional things have to be specified. One is the Descriptor type that tells the framework whether its results will be related to individual objects (object_oriented) or if they will be related to the whole image (image_oriented). This will affect the structure of the resulting data messages. The other, related_to, is the category specified by the detector configuration file.
